# Propagation and Attenuation Characteristics of an Ultrasonic Beam in Dissimilar-Metal Welds

**DOI:** 10.3390/s20216259

**Published:** 2020-11-02

**Authors:** Young-In Hwang, Deokyong Sung, Hak-Joon Kim, Sung-Jin Song, Ki-Bok Kim, Sung-Sik Kang

**Affiliations:** 1 Safety Measurement Institute, Korea Research Institute of Standards and Science, Daejeon 34113, Korea; yihwang@kriss.re.kr (Y.-I.H.); kimkibok@kriss.re.kr (K.-B.K.); 2 Department of Civil & Railroad Engineering, Daewon University College, Jecheon 27135, Korea; 3 School of Mechanical Engineering, Sungkyunkwan University, Suwon 16419, Korea; hjkim21c@skku.edu (H.-J.K.); sjsong@skku.edu (S.-J.S.); 4 Department of Nuclear Safety Research, Korea Institute of Nuclear Safety, Daejeon 34142, Korea; sskang@kins.re.kr

**Keywords:** ultrasonic testing, dissimilar-metal welds (DMWs), ultrasonic beam characteristics, ultrasonic attenuation, anisotropy, beam distortion

## Abstract

Ultrasonic inspection of welds joining dissimilar metals in nuclear power plants has proven to be a challenge, because the ultrasonic waves are subject to diffraction, distortion, scattering, and noise. These perturbations are due to their interactions with coarse-grained microstructures having anisotropic and heterogeneous metallurgical properties that can promote ultrasonic attenuation. In this paper, to improve the reliability of ultrasonic testing for dissimilar-metal welds (DMWs), ultrasonic beam characteristics for DMWs with a buttering layer were investigated in order to analyze the beam distortion phenomenon caused by inhomogeneous anisotropic properties and coarse grains. Ultrasonic testing was performed on DMW specimens using single ultrasonic transducers to investigate the behavior of the ultrasonic beam in the welds. According to the anisotropic and heterogeneous properties, when passing through the weld and the buttering layer of the DMW, ultrasonic waves were distorted and attenuation was high. In particular, in the case of using angular incidence that passed through the weld and the buttering layer in turn, the received ultrasonic data did not contain accurate internal information. From this, it was verified that internal defects may be detected by transmitting ultrasonic waves in different directions. Finally, the existing limitations on the application of non-destructive ultrasonic testing to dissimilar-metal welds were verified, and a solution to the measurement method was proposed.

## 1. Introduction

Welding and joining techniques are very important in nuclear power systems. This is because nuclear installations consist mostly of welded structures involving a wide variety of interconnected materials, and because the welds and joints are usually the most vulnerable areas. Like general industrial facilities, nuclear power plants must be designed and manufactured to combine various kinds of metal materials and connect them by welding, so that the desired functions can safely be performed during the required periods. Although nuclear power plant construction is similar to most general industrial plants in the case of applying welding or joining methods, several differences must be taken into account due to the specificity of the operating conditions of nuclear power plants. Since nuclear power plants use nuclear fuel, many safety systems are needed to prevent damage to the facilities that could cause radiation leakage. Therefore, compared with general industrial plants, the piping system of a nuclear power plant is very complicated and has many welded parts. In addition, so many different materials are welded to each other because these various materials are those suitable for the facility to improve its efficiency and economic feasibility.

In the case of nuclear power plants in operation, high radiation levels are caused by activation from contaminated cooling water or neutron irradiation, so repairing damaged welds is more complicated and difficult than in general industrial plants. In the case of general industrial equipment, the welding operators may approach the welded parts to remove the damaged portion and perform repair welding. However, in the case of nuclear power plants, the welding technicians find access difficult due to the high radiation; thus, automation and remote control of the welding apparatus are required. Of course, it is preferable to prevent the occurrence of damage in the welded parts by satisfying high-quality conditions and thorough welding management in the manufacturing stage. In addition, the welds must be thoroughly managed and maintained in accordance with the procedures established during operation.

Highly toxic additives are used in the cooling water that removes the heat generated by the reactor while the nuclear power plant is operating, so corrosion-resistant materials must be used in the vessel and piping system. Regarding the base materials, it is possible to develop and use materials with improved corrosion resistance at the material manufacturing stage, but in the case of welded parts, it is difficult to obtain the same corrosion resistance as in the base materials. Moreover, the tendency to corrosion that is already characteristic in welds may be worsened by residual stresses formed during the welding of dissimilar materials (called dissimilar-metal welds, DMWs) and during later operations. Therefore, research to improve the physical properties, economics, and reliability of the bonding of dissimilar metals (particularly between low-alloy steel and austenitic stainless steel) utilized in pressurized water reactors (PWRs) has been carried out continuously.

DMW in nuclear power plants is applied to joints between low-carbon-steel containers and stainless-steel pipes, and between low-alloy-steel containers and Inconel alloy penetrations such as Alloy 600 [1,2,3,4,5,6]. Primary water-stress corrosion cracking (PWSCC) may occur on the inner surface of a pipe in contact with the water in the primary cooling system [7]. PWSCC occurs in dissimilar-metal weldment applied to the nuclear power equipment and piping under operating conditions typical of pressurized water reactors. This cracking is generated by defects arising from stress corrosion because of the cracking of hardened oxide layers or arising from environmental conditions such as selective corrosion and inclusion elution. These start as micro-cracks on the surface but can grow into long cracks that are potentially dangerous because of their rapid growth. In fact, because the damage caused by PWSCC frequently occurs in DMW parts applied to reactor penetration pipes, and in other large and small diameter pipes, defect detection technologies able to prevent or solve such problems are necessary.

The methods for inspecting the presence of defects can be divided into two types: destructive and nondestructive methods. Due to the fundamental constraint of destroying structures when applying destructive methods, inspections of operating nuclear power plants have been carried out mainly in nondestructive ways [8,9]. Among them, ultrasonic testing can be mentioned as a more economical technical alternative that also solves the safety management problem that occurs with radiographic testing. In the testing of flaws with ultrasonic waves, which are sound waves above the audible frequencies that can be heard by humans, the waves are transmitted into the weld zone; after which, their characteristics are evaluated by receiving ultrasonic waves reflected from inherent defects. This is very economical, because it incurs little additional cost for repeated tests and is safe. However, for manual ultrasonic flaw testing, which is widely applied in the current industrial field, inspectors analyze the test signals appearing in an ultrasonic flaw detector to determine the presence, location, type, and size of the flaws. For this reason, excessive subjectivity is likely to influence the acquisition of fault signals and interpretation of the results. In addition, the anisotropy of the welded metal causes differences in the acoustic impedance within the grain boundaries, which makes the detection of flaws there difficult because the ultrasonic velocity is changed, the direction is distorted, and the ultrasonic energy is attenuated.

On the other hand, the ultrasonic testing of DMWs requires more consideration than the inspection of other welds. Due to their nonuniform structures, in which the velocity of ultrasonic waves varies depending on the orientation of the crystal structure, the direction is distorted, and scattering occurs when the ultrasonic energy penetrates the weld zone [10,11]. In other words, there is a beam-distortion phenomenon that occurs during defect inspection using the ultrasonic technique. This distortion is caused by the granular crystal grain size, the complex grain size, the local orientation, and the interface of the weld [12,13,14,15]. In addition to these weld characteristics, more inaccurate assessments are caused in detecting defects in the buttering layer in DMWs as applied to nuclear power plants. To overcome such a limitation, studies on the nondestructive evaluation of welds of dissimilar metals using ultrasonic waves have been continuously conducted in recent years. Gezaei Abera et al. measured the grain structure from a photomacrograph of a DMW to find a practical way to predict defects. They also estimated the grain orientation (with the help of a numerical simulation) for a given asymmetric DMW component with specified scatterers. This was done to distinguish defect signals during practical DMW testing and to optimize the signal-to-noise ratio during ultrasonic testing [16]. Ghosh et al. carried out an ultrasonic evaluation of joints to ensure the integrity of welds joining low-alloy steel and austenitic stainless steel. To identify the failure-prone area across the welded joint, the ultrasonic velocity (in longitudinal and shear mode) was measured along the transverse direction of the weld. Young’s modulus was obtained from ultrasonic measurements to determine the yield strength of different regions across the welds [17]. Gardahaut et al. evaluated the propagation of elastic waves in anisotropic and inhomogeneous media using a newly developed ray-tracing algorithm and DMW mock-up with a crystallographic orientation of its constitutive grains [18]. Yin et al. verified the detectability of subsurface planar defects in ferrite-austenite-type dissimilar-metal welds using an angle longitudinal wave technique [19].

DMWs are currently being tested using procedures such as PDI UT-10 [20,21,22], which was validated by the US Electric Power Research Institute (EPRI). However, the shape of the specimens used in US EPRI testing are mostly based on those used in boiling water reactors (BWRs), which have a shape different from the DMWs present in PWRs. A PWR contains pressure vessels and steam generators inside pressure containment vessels. It consists of a primary system where the coolant circulates in the reactor, a secondary system that runs the turbine and generator with hot steam generated from the steam generator, and a tertiary system that turns the steam back into water. These systems include various types of parts joined using DMW in the main equipment of the primary system, which is the reactor coolant system. Moreover, alloy 82/182 [1,23] is mainly used as a filler metal in these parts. This filler metal has a coefficient of thermal expansion between those of low-alloy steel and austenitic stainless steel and has the excellent ability to prevent carbon diffusion from the low-alloy steel to the weld. However, in previous studies, there has been insufficient consideration of the propagation characteristics of ultrasonic waves in dissimilar-metal welds. To apply nondestructive ultrasonic techniques to the DMW parts in a nuclear power plant, it is necessary to check on any constraints applied to them. This is done based on the results obtained by applying the existing experimental techniques to the DMW parts.

In this study, in order to improve the reliability of testing using the ultrasonic flaw detection of DMWs, ultrasonic testing was performed on welds extracted from nuclear power plants. Then, the characteristics of the ultrasonic wave propagation in the welds were analyzed. In addition, the physical properties of the DMWs were measured by calculating the attenuation coefficients for each part of the DMWs, and the characteristics of the reflected ultrasonic signals were analyzed to reveal the internal defects.

## 2. Theory of Ultrasonic Attenuation

Ultrasonic waves propagating inside a material can be used to provide information about the material. The information indicated by the waves is about phase and amplitude, but they can be interpreted to reveal a variety of properties inside the material. When ultrasonic waves are generated and transmitted through the material, energy losses are obvious. With ultrasonic propagation, attenuation occurs due to the effects of scattering, absorption, and the difference in acoustic impedance of two different materials at their interface. The influence of interference is shown by phase shift, frequency shift, wave fringes due to diffraction, and other factors.

**Figure 1:**
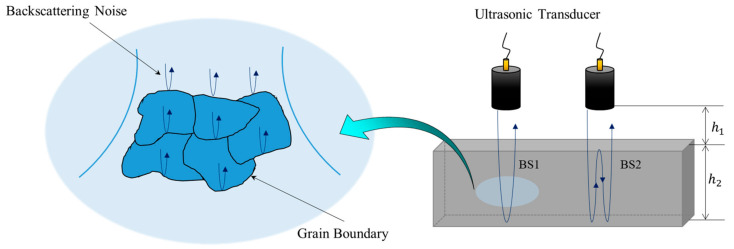
Evaluation of the physical property by backscattering of a transmitted ultrasound.

Among the attenuation factors, scattering occurs because the composition of the material is not completely uniform. Scattering occurs due to the presence of interfaces with different acoustic impedance values (such as inclusions and pores) or due to the grain boundaries within the material. In heterogeneous materials with very different densities and modulus, scattering occurs as ultrasonic waves pass through the material due to the difference in acoustic impedance of each particle in the material. Even with a single-crystal structure, when the crystalline parts of the material are anisotropic, the random directionality of each grain in the material causes scattering (as in other materials composed of crystals). In particular, with the ultrasonic inspection of structures made of anisotropic media, such as DMWs, there are many difficulties in detecting internal defects, because backscattering noise caused by internal grain boundaries is relatively higher than that of isotropic media. Figure 1 illustrates these characteristics of a transmitted ultrasonic beam.

In general, because the attenuation of ultrasonic waves depends on their frequency, the ultrasonic attenuation per unit distance—that is, the attenuation coefficient—also depends on the frequency of the ultrasonic waves. The decrease in sound pressure due to attenuation by scattering and absorption can be expressed as an exponential function, as shown in Equation (1).

(1) $$\frac{P}{P_{0}}=e^{-\alpha \left(f\right)d}$$

where $P_{0}$ is the initial sound pressure at d=0, P is the sound pressure at distance d, α is the attenuation coefficient at frequency f, and d is the traveling distance of the ultrasonic wave in the material.

Ultrasonic signals received by reflection back to the bottom of the test object using the pulse-echo method gain information about the physical properties of the subject as the ultrasound passes through the material in a reciprocating way. Using this principle, the attenuation coefficient of the material can be calculated through multiple back echoes. The multiple back echoes obtained can be used to calculate the attenuation coefficient, as indicated in Equation (2) [15,24].

(2) $$\alpha_{s}\left(f\right)=\frac{1}{2h_{2}}ln\left|e^{2ik_{2}h_{2}}\frac{{\left(R_{21}^{P;P}\right)}^{2}D_{P}\left(\frac{k_{p1}a^{2}}{2D_{bs2}}\right)}{D_{P}\left(\frac{k_{p1}a^{2}}{2D_{bs1}}\right)}\frac{V_{BS1}\left(\omega \right)}{V_{BS2}\left(\omega \right)}\right|$$

where $\alpha_{s}$ is an attenuation coefficient, $V_{BS1}$ is the first backwall echo, and $V_{BS2}$ is the second backwall echo. DP($k_{p1}a^{2}$/$\left(2D_{bs2}\right))$ and DP($k_{p1}a^{2}$/$\left(2D_{bs1}\right))$ are the diffraction corrections, where $D_{bs1}$ and $D_{bs2}$ are the distance from transducer to the first backwall echo and the second backwall echo, respectively. Here, a is the radius of the transducer, k is the wave number, $\alpha_{w}$ is the attenuation of water, $h_{1}$ is the water path, and $h_{2}$ is the thickness of the specimen. The terms $V_{BS1}$ and $V_{BS2}$ can be expressed as:

(3) $$V_{BS1}\left(\omega \right)=\beta \left(\omega \right)T_{12}^{P;P}R_{21}^{P;P}T_{21}^{P;P}e^{2ik_{1}h_{1}}e^{2ik_{2}h_{2}}e^{-2\alpha_{w}h_{1}}e^{-2\alpha_{s}h_{2}}D_{P}(\frac{k_{p1}a^{2}}{2D_{bs1}})$$

(4) $$V_{BS2}\left(\omega \right)=\beta \left(\omega \right)T_{12}^{P;P}R_{21}^{P;P}R_{21}^{P;P}R_{21}^{P;P}T_{21}^{P;P}e^{2ik_{1}h_{1}}e^{4ik_{2}h_{2}}e^{-2\alpha_{w}h_{1}}e^{-4\alpha_{s}h_{2}}D_{P}(\frac{k_{p1}a^{2}}{2D_{bs2}})$$

where $\beta \left(\omega \right)$ is the system efficiency factor, and $T_{12}$ and $T_{21}$ are the transmission coefficients between water and the test specimen. $R_{12}$ is the reflection coefficient and can be expressed as in Equation (5):

(5) $$R_{12}=\frac{\rho_{s}c_{s}-\rho_{w}c_{w}}{\rho_{w}c_{w}+\rho_{s}c_{s}}$$

where $\rho_{w}$ and $\rho_{s}$ are the density of the water and test specimen, and $c_{w}$ and $c_{s}$ are the sound velocities for water and the test specimen.

## 3. Propagation Characteristics of Ultrasound Transmitted into DMWs

### 3.1. Fabrication of Coupon Samples

**Figure 2:**
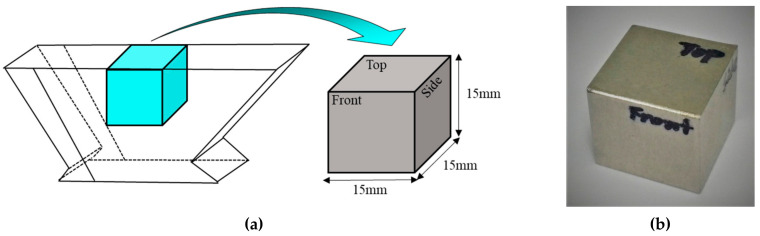
(**a**) Schematic illustration and (**b**) photograph of a coupon specimen of weld metal.

Test coupons were fabricated from weldment parts in the DMWs to analyze the propagation characteristics and beam splitting of the ultrasonic waves. Figure 2 shows a 15-mm cube coupon sample composed only of filler-metal material from a DMW.

### 3.2. Ultrasonic Characteristics of a Coupon Sample

**Figure 3:**
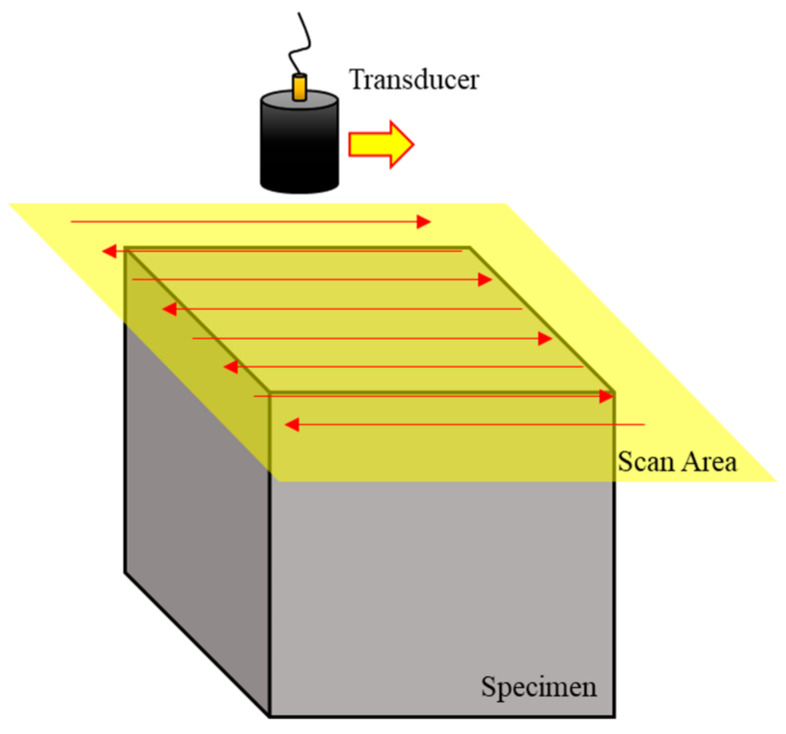
Experimental setup for the coupon specimen using the pulse-echo method.

First, ultrasonic immersion testing was carried out, in which ultrasonic waves were vertically directed toward the top, front, and side planes using the ultrasonic pulse-echo method. The water distance was set to 12.25 mm, and a 6.35-mm diameter circular plane transducer with a 5-MHz frequency was used. The −6 dB fractional bandwidth was 85%. As shown in Figure 3, scanning was performed at 0.5-mm intervals over a 25-mm square area (wider than the specimen surface). C-scan images were depicted using signals obtained by the reflection of each plane while parallel to each of the three planes in the sequence, because the most suitable result for analyzing ultrasonic wave propagation can be obtained from the C-scan image.

**Figure 4:**
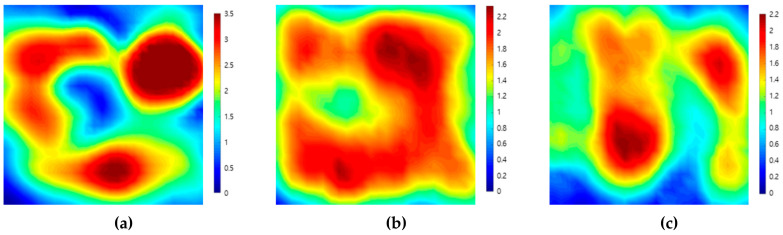
Ultrasonic images acquired by scanning the (**a**) top, (**b**) front, and (**c**) side planes of a coupon. Specimen using the first back-surface echo and the ultrasonic pulse-echo method.

**Figure 5:**
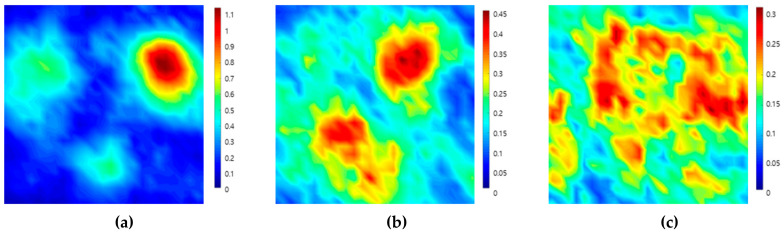
Ultrasonic images acquired by scanning the (**a**) top, (**b**) front, and (**c**) side planes of a coupon specimen using the second back-surface echo and the ultrasonic pulse-echo method.

Figure 4 shows the C-scan images depicted by ultrasonic signals transmitted toward the top, front, and side planes of the filler-metal coupon. Since the highest amplitude values in each result were different, color map ranges suitable for each image were applied. It can be seen that the results on the three sides are different depending on the grain orientation of the weld. The reason for this result is that the sound propagation was affected by the ultrasonic deviation. This is because there were coarse grains larger than the wavelength in the weld metal of the sample. Figure 5 shows the C-scan images depicted using the second back-surface echo acquired by the ultrasonic transmission to the top, front, and side planes of the filler-metal coupon. As the incident ultrasonic wave passes through the filler-metal up and down twice, it can be seen that the signal amplitude is reduced due to the attenuation effect. Based on an analysis of the images depicted using the first backwall echo, the results of the ultrasonic testing performed on the weld were considered to increase the difficulty in interpreting the ultrasonic signals due to the same factors.

**Figure 6:**
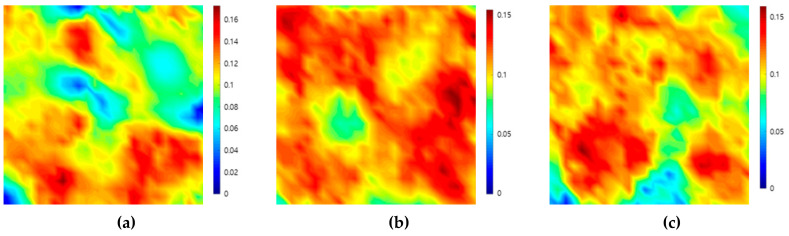
Ultrasonic attenuation map from the (**a**) top, (**b**) front, and (**c**) side scanning of a weld-metal specimen using attenuation coefficient values and the ultrasonic pulse-echo method.

By the signals used to depict each of the images shown in Figure 4 and Figure 5, the attenuation map for the sample can be depicted by calculating the attenuation values for each position. Figure 6 shows the ultrasonic attenuation map depicted using the attenuation coefficients obtained from the first back-surface echo and the second back-surface echo by scanning the top, front, and side planes of the filler-metal sample. Through these images visualized using values calculated quantitatively using Equation (2), it can be seen that the color differences of each position shown in the images reflect the attenuation effect as the ultrasound wave reciprocates up and down twice within the filler-metal.

Since the attenuation is affected by the frequency of the transducer used, there will be a difference in the attenuation values when using a transducer of a different center frequency. Accordingly, the color difference in the image will appear differently, but it is thought that it will show a similar tendency.

**Figure 7:**
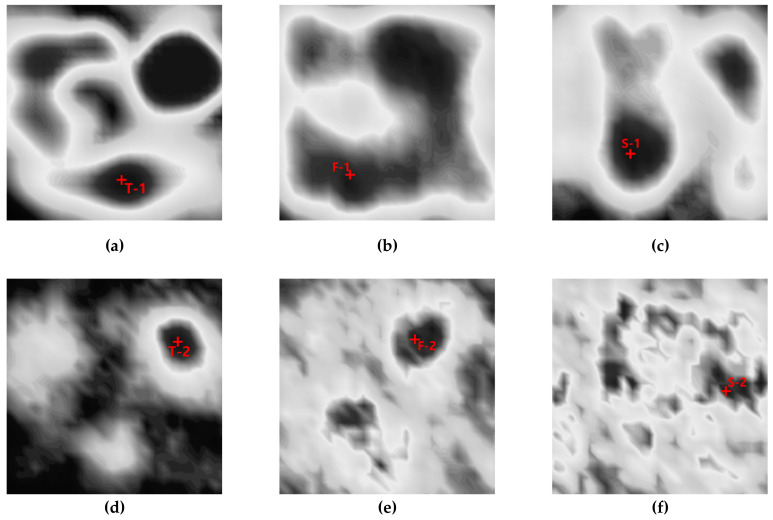
C-scan images with points of the highest amplitude as determined by the first back-surface echo obtained from scanning toward the (**a**) top, (**b**) front, and (**c**) side planes and by the second back-surface echo obtained from scanning toward the (**d**) top, (**e**) front, and (**f**) side planes.

To verify the attenuation effect on each plane, attenuation coefficients were calculated for the locations with the largest signal amplitudes in the two sections where the first and second back echoes (respectively) were strong. Figure 7 shows the two positions selected for each plane, and Table 1 shows the attenuation coefficients for each plane, as calculated using Equation (2).

**Figure 8:**
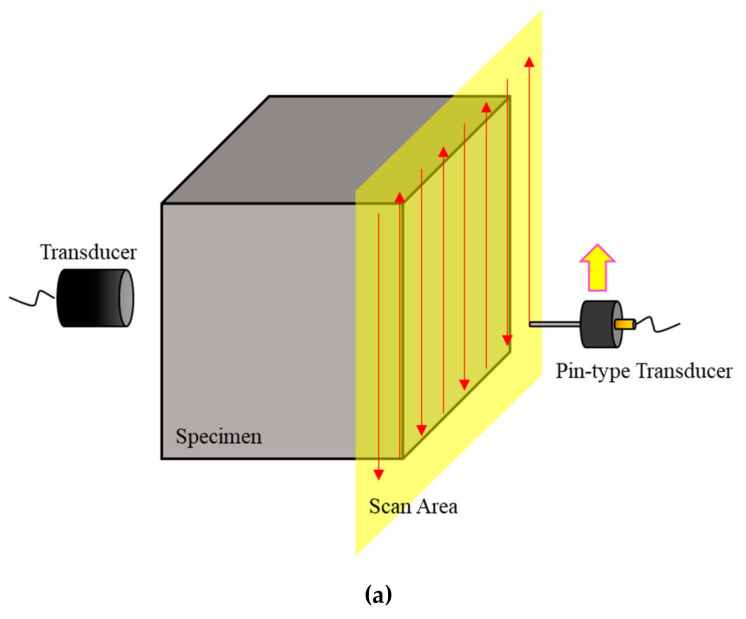

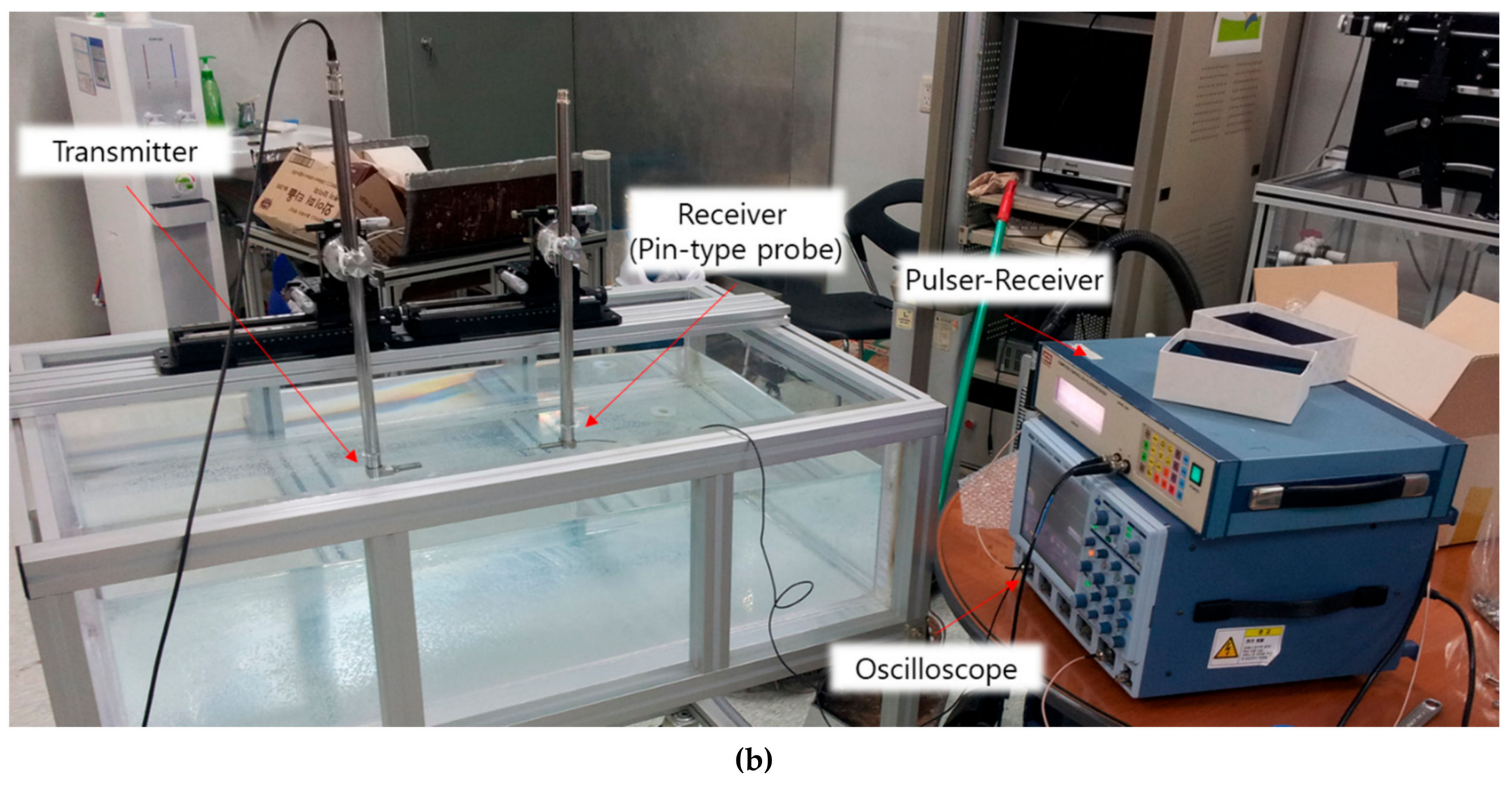
(**a**) Schematic illustration and (**b**) photograph of the experimental setup for the coupon specimen using the through-transmission method.

An experiment was also performed in which a through-transmission method was performed using a pin-type transducer. As shown in Figure 8, the same transducer used in the experiment with the pulse-echo method was used as the transmitter. Its position was fixed at a distance of 12.25 mm vertically from the center of the scan area. A transmitted ultrasound was passed through the test subject, and the signal was acquired by the pin-type transducer. The diameter of the pin-type transducer was 2.413 mm and its central frequency 5 MHz. The −6 dB fractional bandwidth was 78%. The location of the pin-type transducer was changed and scanned using the same area and step as in the experiment using the pulse-echo method. This experiment was also performed on three sides and at the same distance as in the experiment in which the pulse-echo method was applied.

**Figure 9:**
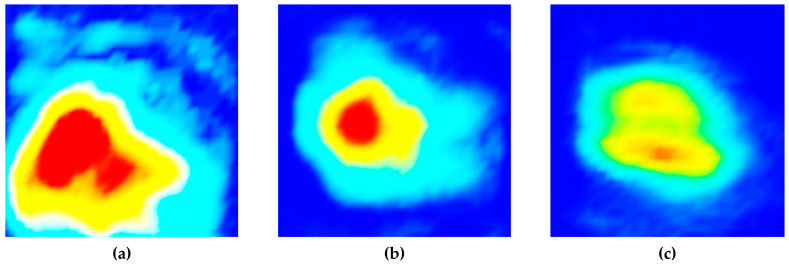
Ultrasonic images acquired by scanning the (**a**) top, (**b**) front, and (**c**) side of a weld-metal specimen using the through-transmission method.

Figure 9 shows the images depicted using the ultrasonic signals acquired from the pin-type transducer by applying the through-transmission method to the coupon sample. The results obtained indicate that the images can be depicted differently, because the beam deviates from the expected propagation direction in the anisotropic weld metal. Accordingly, it was verified that distorted images of the welded part occurred not only in the results from the pulse-echo method (using signals reflected on the bottom surface of the test object), but, also, in the results from the through-transmission method (using signals returned to the receiver after ultrasonic waves were transmitted).

### 3.3. Fabrication of the DMW Specimen

**Figure 10:**
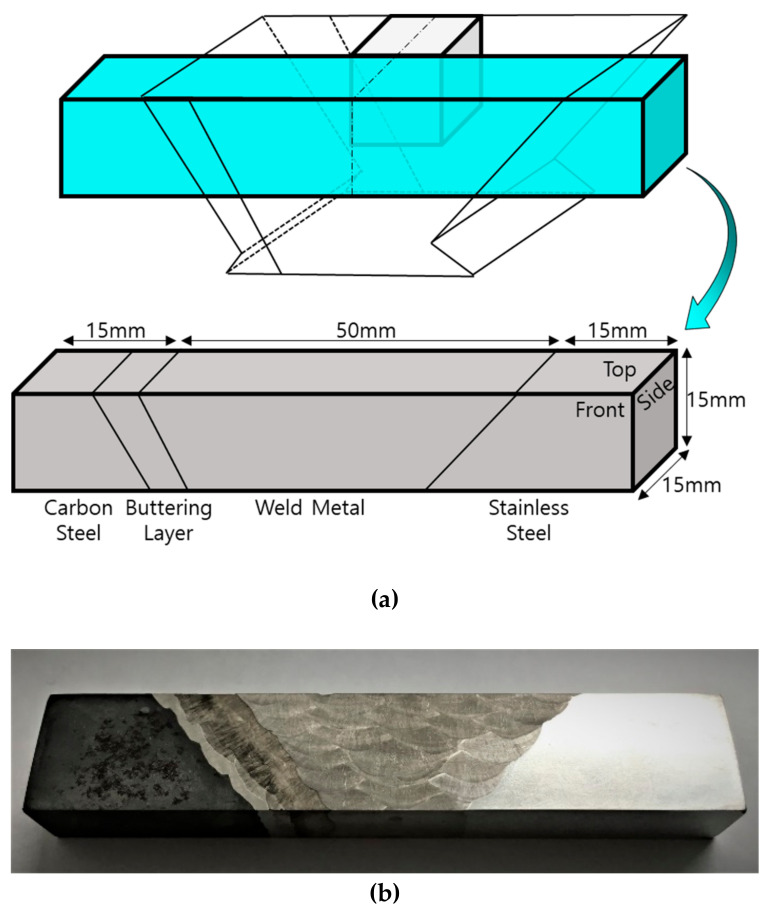
(**a**) Schematic illustration and (**b**) photograph of the dissimilar-metal welds (DMW) specimen.

To analyze the propagation characteristics and beam splitting of ultrasonic waves in the DMWs, a cuboid specimen containing carbon steel, the buttering layer, filler metal, and stainless steel was fabricated. This specimen was fabricated to have the same central axis as that of the coupon sample. The bottom, width, and length of the base of the cuboid was 80 mm, 15 mm, and 15 mm, respectively. Figure 10 includes a schematic illustration showing the dimensions of the fabricated DMW specimen and a photograph of the DMW specimen.

### 3.4. Ultrasound Characteristics of the DMW Specimen

**Figure 11:**
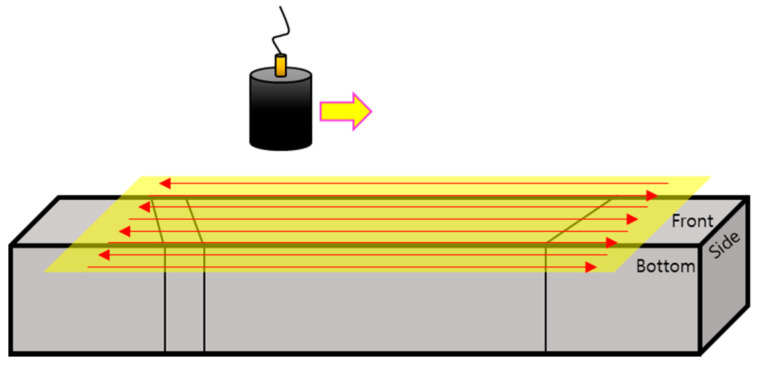
Experimental setup for scanning the dissimilar-metal-welded specimen using the pulse-echo method.

An experiment was performed to depict images of a rectangular parallelepiped specimen having all parts of the DMW. First, immersion testing was carried out perpendicular to the front plane of the specimen using the pulse-echo method on the cuboid, as shown in Figure 11. The scanning area was set to 60 mm × 25 mm to scan all parts of the DMW. The scanning step was 0.5 mm, and the water distance was 17.4 mm. The ultrasonic wave was incident vertically using the same transducer, and the C-scan image was depicted using signals obtained by reflection from the back side.

**Figure 12:**
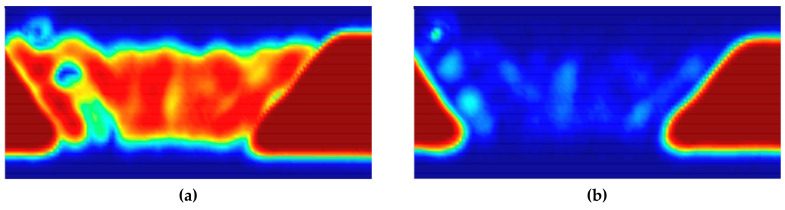
Ultrasonic images acquired by scanning the front plane of the dissimilar-metal-welded (DMW) specimen using (**a**) the first back-surface echo and (**b**) second back-surface echo through the ultrasonic pulse-echo method.

**Figure 13:**
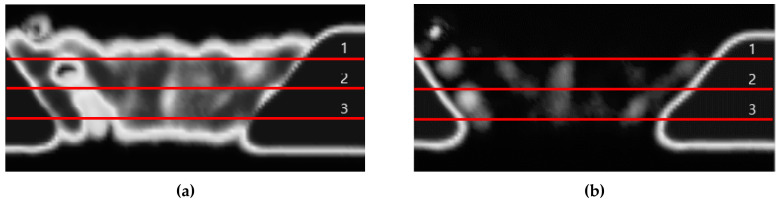
Ultrasonic images by (**a**) the first back-surface echo and (**b**) second back-surface echo with three horizontal lines divided into four equal intervals.

**Figure 14:**
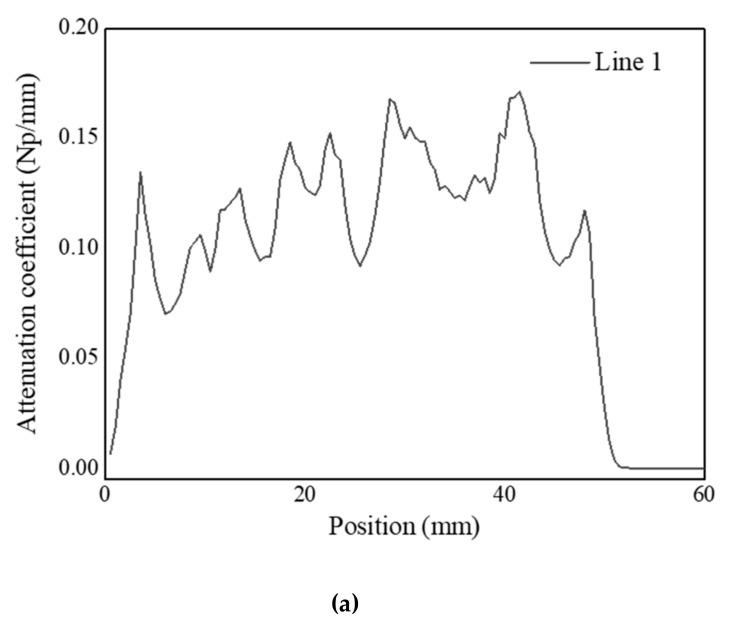

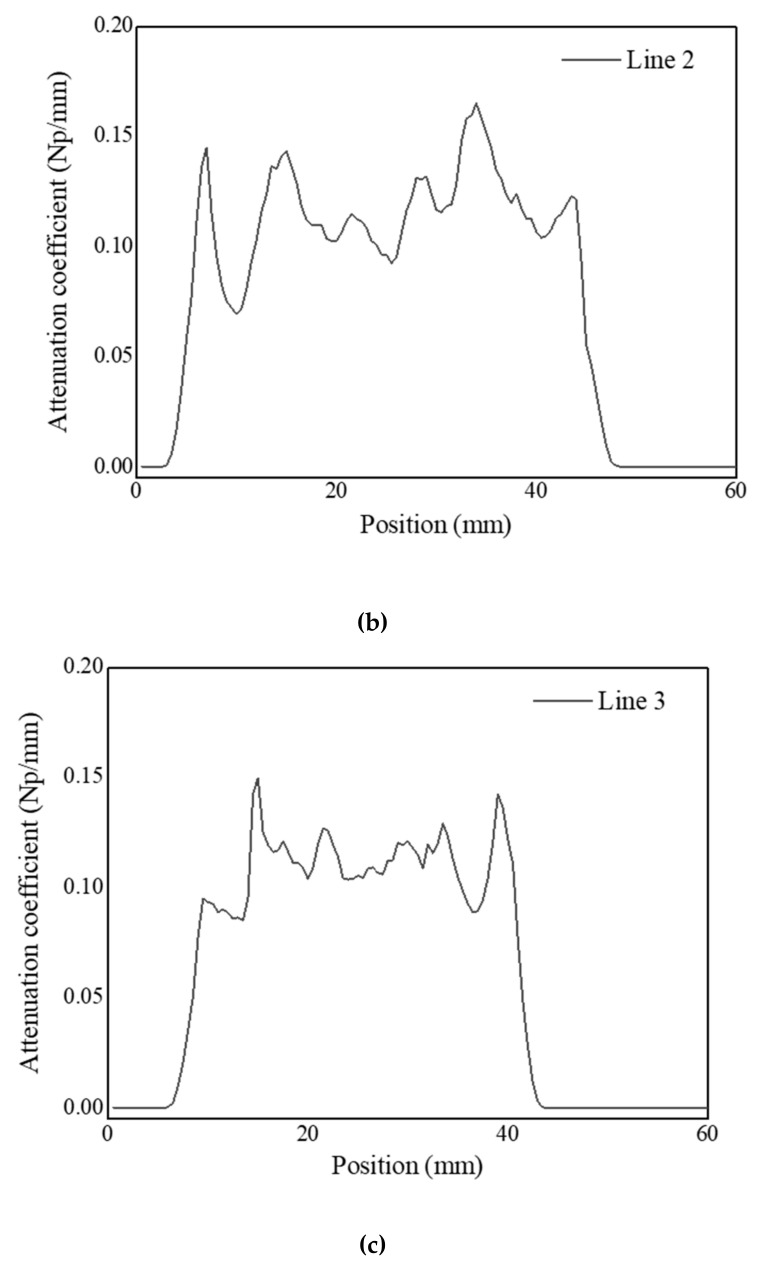
Attenuation coefficients calculated from ultrasonic signals obtained from (**a**) Line 1, (**b**) Line 2, and (**c**) Line 3.

Figure 12 shows C-scan images depicted using the pulse-echo method for the DMW specimen. The C-scan image created by the first back-surface echo was well-identified to the boundary of each part, whereas the images of the buttering layer and weld metal were not properly realized by the second back-surface echo. This is the result of beam attenuation due to the heterogeneity and anisotropy of the buttering area and weld metal. Moreover, amplitude values were plotted along three lines traversing the images of the specimen in the horizontal direction. Figure 13 shows the positions of the lines. The attenuation coefficients were calculated (as shown in Figure 14) using the values obtained along each line. In this experiment, because the attenuation in the stainless steel and carbon steel regions was not very large, it was excluded from the analysis. In order to clearly receive the reflected signals from the weld parts, the gain value was increased to obtain the ultrasonic signals. As a result, the ultrasonic signals received in the stainless-steel and carbon-steel regions were saturated, and accordingly, regions with values of 0 appeared to the left and right of each graph. Compared with the attenuation values of the welds measured in other studies, the attenuation measured in the buttering layer of the specimen used in this study was relatively lower, and the value measured in the weld-metal part was relatively higher.

## 4. Ultrasonic Signal Characteristics of a DMW with Cracks

### 4.1. Fabrication of a Test Specimen with Artificial Cracks

**Figure 15:**
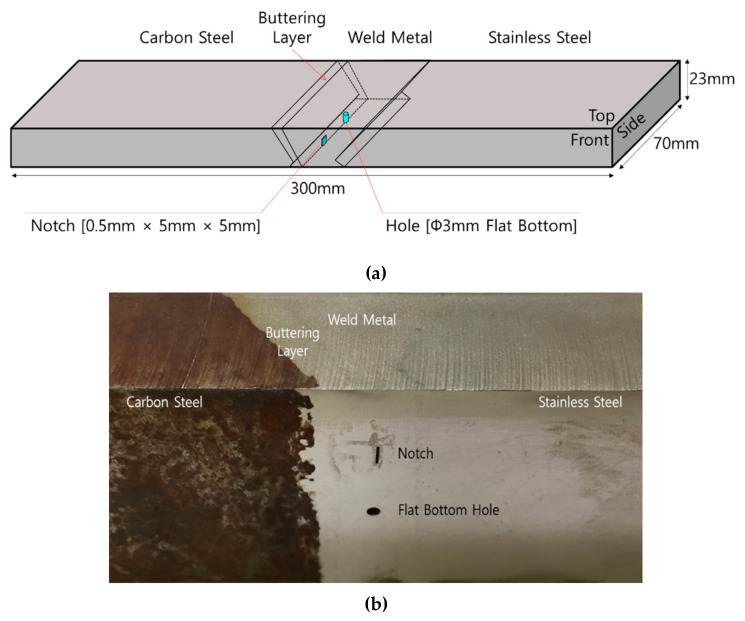
(**a**) Schematic illustration and (**b**) photograph of a DMW specimen with artificial cracks.

In order to perform crack detection in DMWs, another DMW specimen was fabricated. To join stainless steel and carbon steel, a test piece was created by welding Inconel 182 [1] to carbon steel. The test piece was 300-mm-long, 70-mm-wide, and 23-mm-high. Primary water stress corrosion cracking is most likely to grow as the roots crack at the bottom of a weldment [25,26] so a flat bottom hole having a diameter of 5 mm and a notch of 0.5 mm × 5 mm × 5 mm were machined at the boundary between the weld-metal part and buttering layer of the specimen. This boundary is an area where PWSCC is formed and is vulnerable to growth. Figure 15 shows a DMW specimen with artificial defects. 

### 4.2. Ultrasonic Testing for Detecting Cracks in DMW

**Figure 16:**
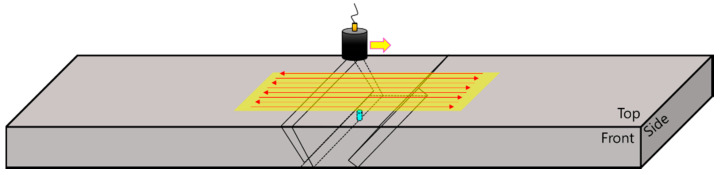
Experimental setup for detecting a crack in a welded specimen of different metals by ultrasonic vertical incidence.

First, C-scan images of the flat bottom hole in the specimen were depicted by transmitting ultrasonic waves vertically. As shown in Figure 16, an area 30 mm × 10 mm around, the flat bottom hole was scanned while immersed using the pulse-echo method. The probe was a 6.35-mm-diameter circular plane transducer with a frequency of 5 MHz, as in the previous experiments.

**Figure 17:**
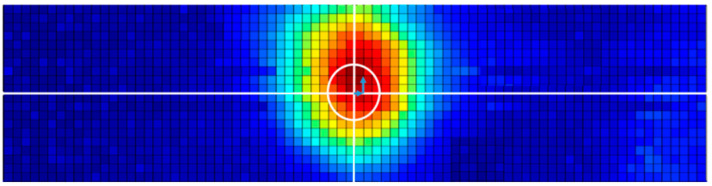
C-scan image of the flat bottom hole in the dissimilar metal weldment specimen.

Figure 17 shows an image of the flat bottom hole obtained by scanning the area of the sample around the flat bottom hole with ultrasonic waves directed downward from above. The white circle in the center of the image represents the diameter of the actual flat bottom hole. Although the ultrasonic wave transmitted to the weld-metal part in the DMW was attenuated, it was possible to identify the crack in the C-scan image from signals reflected at the upper end of the flat bottom hole. However, it was difficult to accurately depict the shape of the actual crack due to the distortion of the ultrasonic beam.

**Figure 18:**
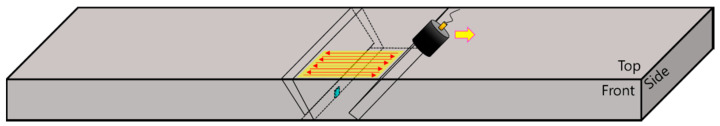
Experimental setup for detecting a defect in the DMW specimen by ultrasonic angular incidence through stainless steel.

Subsequently, an experiment was performed in which the transmitted ultrasonic waves were incident at an oblique angle to detect the notch in the specimen. In accordance with the properties of DMWs (welds of two different base materials), ultrasonic waves were incident in both directions and penetrated each metal. The refraction angles of the ultrasound were set at 45° and 60°. The same transducer was used, and the scanning area was 15 mm × 15 mm. As shown in Figure 18, the experiment was performed for the case in which ultrasonic waves penetrated stainless steel to reach the weld-metal part.

**Figure 19:**
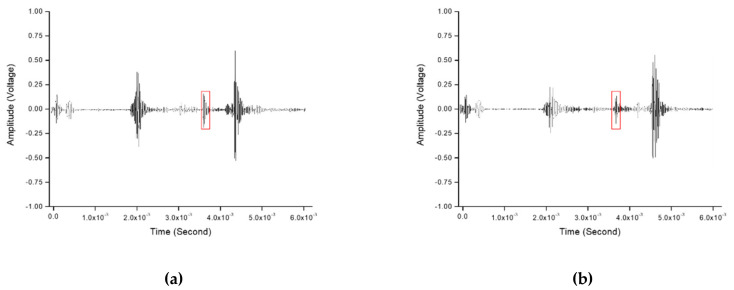
Ultrasonic signals of a dissimilar-metal weldment specimen with notch using (**a**) a 45° oblique incidence and (**b**) 60° oblique incidence through stainless steel.

**Figure 20:**
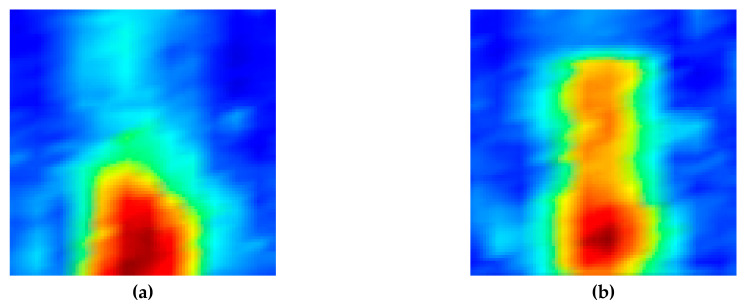
Ultrasonic images of a dissimilar-metal weldment specimen with notch by (**a**) a 45° oblique incidence and (**b**) 60° oblique incidence through stainless steel.

Figure 19 shows the ultrasonic signals obtained experimentally to detect the crack in the DMW by tilting the transducer at 45° and 60° in the direction through the stainless steel, and the crack signals are shown in the section marked with red borders. Through this result, it was possible to verify visually that the defect signals appear clearly at the expected position. Figure 20 shows images obtained experimentally for detecting cracks in the DMW after the incident ultrasound waves were inclined at 45° and 60° in the direction through the stainless steel. In the ultrasonic images depicted by receiving the ultrasonic signals reflected from the artificial crack and obliquely incident through the stainless steel part, the defect images due to the notch were depicted. 

**Figure 21:**
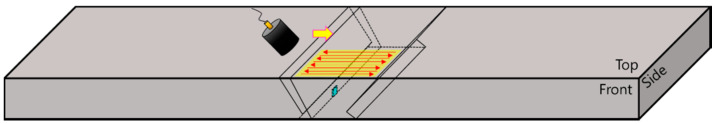
Experimental setup for detecting defects in welded specimens of different metals using ultrasonic angular incidence through the carbon steel and buttering layer.

**Figure 22:**
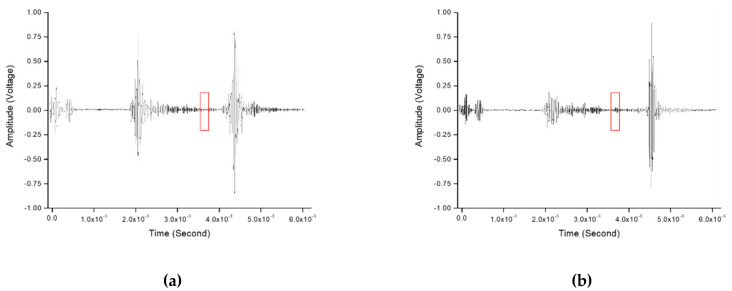
Ultrasonic signals in a dissimilar-metal weldment specimen with a notch using (**a**) a 45° oblique incidence and (**b**) 60° oblique incidence through carbon steel and the buttering layer.

**Figure 23:**
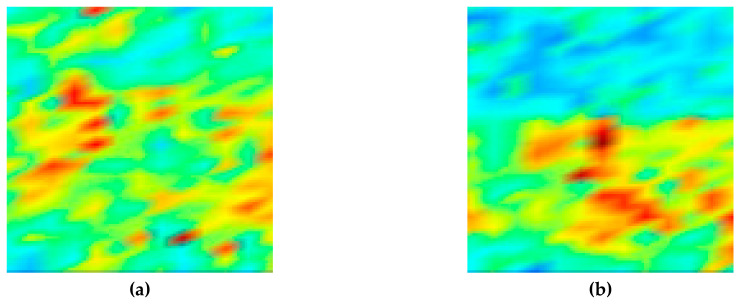
Ultrasonic images of a dissimilar-metal weldment specimen with a notch using (**a**) a 45° oblique incidence and (**b**) 60° oblique incidence through carbon steel and the buttering layer.

Subsequently, experiments were performed in which the ultrasonic waves were passed through the carbon steel and then the buttering layer in turn, as shown in Figure 21. Figure 22 shows ultrasonic signals obtained through the experiments of detecting the crack in DMW by tilting the beam at 45° and 60° in the direction passing through carbon steel and the buttering layer, and the section marked with red borders indicates the location where the crack signals should have appeared. Unlike the ultrasonic signals shown in Figure 19, no crack signal was identified. Figure 23 shows the images obtained through the experiment of detecting the crack in DMW by tilting at 45° and 60° in the direction passing through carbon steel and the buttering layer. The results of the experiments performed by inclined incidence through the carbon steel part showed that the beam distortion phenomenon was severe as the ultrasonic wave reciprocated within the buttering layer and the weld part, because the attenuation value is large in the buttering layer. When detecting defects in DMWs with a buttering layer, the result may vary greatly depending on the direction of the ultrasonic wave transmission. In addition, when the ultrasonic wave passes through the buttering layer, the signal-to-noise ratio may be lower than that of the ultrasonic wave through the stainless steel layer. Accordingly, in order to detect defects in the dissimilar-metal welding parts in a nuclear power plant, ultrasonic waves must be incident in a direction that does not pass through the buttering layer so that the attenuation is relatively low.

## 5. Conclusions

In this paper, a study on the use of a nondestructive ultrasonic diagnosis to evaluate dissimilar-metal welds (DMWs) in nuclear power plants was described. For this purpose, test specimens of different-metal welds (including the buttering layer) were fabricated, and the specimens were used in the analysis of ultrasonic wave characteristics using single ultrasonic probes. The acoustic attenuation coefficients were calculated to evaluate the physical properties of the structure according to the DMW tested. In addition, artificial cracks were created at locations where defects occur most frequently in DMWs, and the ultrasonic signals reflected by the cracks were analyzed.

According to the anisotropic and heterogeneous properties, when passing through the weld and the buttering layer of the DMW, ultrasonic waves were distorted and attenuation was high. As a result, the transmitted ultrasonic energy was diminished so that the reflected signals received, and the ultrasonic image depicted using the signals, did not appear very clear. In particular, in the case of using angular incidence that passed through the weld and the buttering layer in turn, the received ultrasonic data did not contain accurate internal information. From this, it was verified that internal defects may be detected by transmitting ultrasonic waves in different directions.

However, depending on the conditions of the facility structure and site, there may be restrictions when applying ultrasonic testing for crack detection. For example, it is difficult to detect cracks in DMWs using a single probe, so other methods are needed to solve this problem. To minimize the beam-splitting phenomenon of ultrasonic waves transmitted to the test object, applying phased array ultrasonic testing to focus the ultrasonic energy needs to be considered to improve the capability for detection of the cracks in DMWs. 

## Figures and Tables

**Table 1:** Attenuation coefficients at each point calculated from ultrasonic signals obtained by the scanning of each surface.

**Attenuation Coefficient** **(Np/mm)**	**T-1**	**T-2**	**F-1**	**F-2**	**S-1**	**S-2**
	0.112	0.063	0.120	0.088	0.128	0.067
